# Conservative Approach for Traumatic Anterior Crown–Root Fractured Teeth by Orthodontic Extrusion using Customized Mini-Tube Appliance: A Clinical Review

**DOI:** 10.1155/2023/7911464

**Published:** 2023-09-12

**Authors:** Sehun Lim, Youngmin Kwon

**Affiliations:** Department of Conservative Dentistry, Wonju Severance Christian Hospital, Yonsei University, Wonju, Gangwondo, Republic of Korea

## Abstract

A complicated crown–root fracture is a fracture involving enamel, dentin, cementum, and pulp. Because crown fracture generally extends below the gingival margin, several options may be indicated to expose the margins before permanent restoration. Among them, orthodontic extrusion is the most non-invasive treatment option. In this case report, a case of traumatic crown–root fracture of the maxillary incisor was managed by root canal treatment with fiber-reinforced ceramic post-placement followed by orthodontic extrusion using a customized mini-tube appliance technique. Then, the porcelain fused zirconia crown was restored. Traumatized orthodontic extruded teeth have shown a reliable prognosis without inflammatory signs nor complications after a 15-month follow-up.

## 1. Introduction

Trauma-related tooth fracture has a prevalence of 17.5%, of which the crown–root fracture is approximately 5%, which is typically caused by direct trauma to the anterior teeth [1, 2]. Crown–root fractures include fractures that spread to enamel, dentin, and cementum and those that involve pulp exposure and those that do not. However, when the tooth is fractured to the periodontium or below the alveolar bone, such as the crown–root fracture, the restorative treatment is particularly difficult [3]. To restore crown–root fractured teeth, maintaining the biological width of the teeth is an important factor. If the fracture line is in a range that does not invade the biological width of the teeth, fragment reattachment may be possible. However, if the planned restoration is at risk of invading the biological width of the teeth, a clinical crown lengthening procedure should be considered. Biological width has been mentioned by Gupta et al. [4], and it is the width required to defend against bacterial infection from the gingival sulcus to periodontal tissue [5]. Therefore, ensuring that restorative treatment does not destroy or mechanically invade this biological width is important. Methods of increasing the biological width include alveolar bone graft, apically positioned flap operation, surgical extrusion, or intentional replantation [2, 6, 7].

In cases where the dentogingival complex and keratinized gingiva are sufficient, considering that soft tissues are sufficient, treatment can be performed only by gingivectomy through an external incision. However, regardless of the amount of dentogingival complex, if keratinized gingiva is as insufficient as <2 mm, a gingivectomy may not be possible and an apically positioned flap operation must be performed [29]. Such forms of the surgical crown lengthening procedure are not recommended in aesthetically important areas, including maxillary anterior teeth; thus, the acquisition of biological width through surgical extrusion or orthodontic extrusion is recommended. The first objective of surgical extrusion or orthodontic extrusion is to provide sound periodontal tissue at the time of final restoration and to ensure biological width so that the patient can easily maintain and care for the teeth [8]. Surgical extrusion can be performed promptly and simply by dislocating the tooth and extracting it to the desired position after confirming the relationship between the fracture line of the tooth and the bone. However, one demerit includes the possibility of damage to the periodontal ligament cells during the treatment. A more conservative method may be opted for orthodontic extrusion. This treatment is a technique that can be selected from aesthetically important teeth and is recommended first as it provides a better crown-to-root ratio and more aesthetic results than surgical procedures alone [4]. Orthodontic extrusion can typically be performed using removable appliances and rubber bands or brackets and wires [9]; however, these conventional methods have problems, such as possible discomfort during treatment, difficulty in oral hygiene management, and the need for cooperation [3].

To overcome the shortcomings of the existing methods, mini-tube appliance (MTA), typically used for partial correction of anterior tooth, can be used. The MTA uses a mini tube about 3 mm in length, with almost no foreign body sensation or pain, more aesthetic with a lower dropout rate, and the correction period is short, as early as 6 weeks, with an average of 3–6 months [10, 11]. In addition, one of the advantages includes the low frictional resistance, which ensures that it can move quickly with only the light force of wire [10]. In this case, a customized mini tube was used, and a syringe needle tip cut into 3 mm lengths was used as a tube substitute, for easy use. The following case report, in which a patient obtained conservative and aesthetic treatment results using orthodontic extrusion with a customized mini tube for complex crown–root fracture of maxillary incisors due to trauma, and considerations during the procedure were reported.

## 2. Case Report

**Figure 1:**
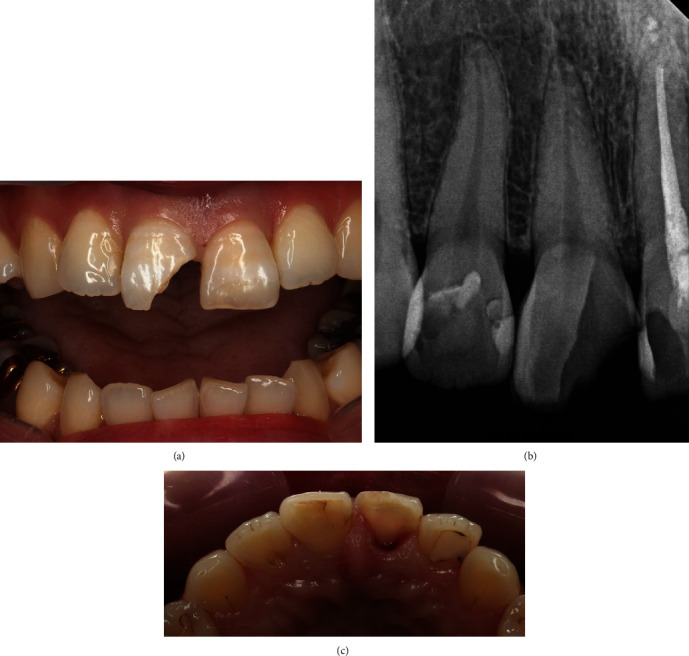
(a) Intra-oral photograph (Buccal), (b) Periapical view, (c) Intra-oral photograph (Occlusal) at first visit.

A 39-year-old woman who fell at the apartment stairs has visited our emergency center. An oral and maxillofacial surgeon sutured the lacerated upper lip and referred her to the Department of Conservative Dentistry, for the treatment of fractured teeth. A complicated crown fracture was visible in the maxillary right central incisor (tooth #11), and an uncomplicated crown fracture was observed in the maxillary left central incisor (tooth #12). The fracture line of tooth #11 was located in the 1.5 mm sub-gingival level on the mesial side. The fracture line of tooth #12 was located on the incisal level (Figure 1).

**Figure 2:**
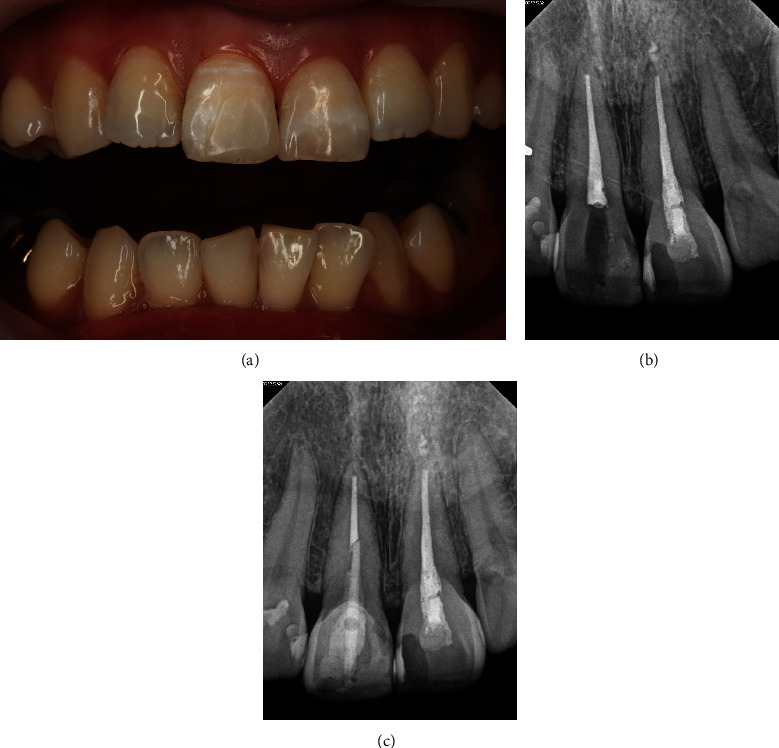
(a) Intra-oral photograph after post placement and resin build-up. (b) Periapical view after canal filling. (c) Periapical view after post placement, and resin build-up.

**Figure 3:**
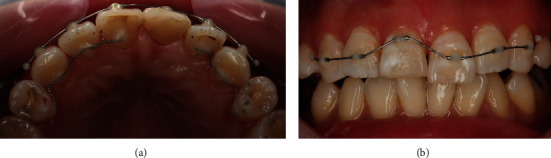
The clinical procedure of customized resin tube appliance. Intraoral photographs of (a) Palatal fixed retainer attachment. (b) Formation of a customized resin tube for orthodontic extrusion procedure.

The treatment plan was to perform porcelain fused zirconia crown restoration on tooth #12 and orthodontic extrusion on tooth #11 after root canal treatment with post-placement due to 1.5 mm sub-gingival leveled crown–root fracture followed by porcelain fused zirconia crown restoration. Root canal treatment under rubber dam isolation was performed first, followed by fiber-reinforced ceramic post (Luxapost, 1.35 mm, DMG, Hamburg, Germany) placement, during which the tooth was etched with 37% phosphoric acid, rinsed, and dried. Then, the bonding layer (All-Bond Universal, BISCO, Schaumburg, IL, USA) was applied, and post-cementation was performed using dual-cure resin cement (Duo-Link Universal, BISCO; Figure 2) Since the buccal surface of residual crown height was long enough, it was not suitable for forced eruption using a hook. Thus, customized MTA was made using a 3 mm length syringe tip for orthodontic extrusion. Maxillary six incisors were prepared with 37% phosphoric acid etching, rinsed, and dried followed by bonding layer (All-Bond Universal, BISCO) application. The MTA was bonded to the central part of the labial surface using flowable composite (Supreme Flowable Restorative, 3M ESPE, St. Paul, MN, USA) so that the NiTi wire could be passively inserted into the maxillary incisors except for the traumatized tooth. MTA of tooth #11 was positioned 1.5 mm to the apical side so that the extrusion force could be only applied to tooth #11. A 012 NiTi wire (HUBIT, Anyang, Korea) was inserted to provide as weak and sustained force as possible, and a palatal fixed retainer was bonded with 0195 twistflex wire (Dentaflex, DENTAURUM, Ispringen, Germany) to the palatal surface of maxillary incisors except tooth #11 to prevent adjacent teeth from shifting (Figure 3).

**Figure 4:**
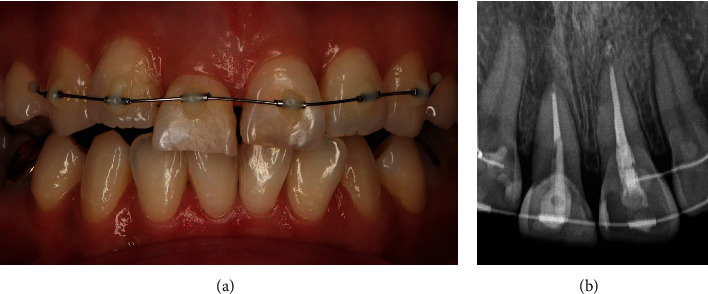
(a) Intra-oral photograph of 4 weeks after orthodontic extrusion started. (b) Periapical view of 4 weeks after orthodontic extrusion started.

**Figure 5:**
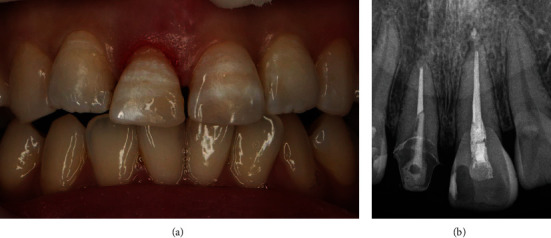
(a) Intra-oral photograph of orthodontic extrusion finished followed by gingivectomy. (b) Periapical view after orthodontic extrusion finished and temporary crown delivery.

Two weeks after orthodontic extrusion started, tooth #11 extruded 1.5 mm to the coronal axis observed. Thus, occlusal reduction and re-adjustment of tooth #11 were performed twice at intervals of 2 weeks. Orthodontic extrusion force was applied for a total of 4 weeks to extrude 3.0 mm length, thereby exposing the fracture line on the mesial side to the supra-gingival level to secure ferrule for dentin. Periapical radiographs also showed that tooth #11 was shifted towards the incisal margin (Figure 4). A gingivectomy with no bone remodeling was also performed to excise the proliferated gingival tissue when the orthodontic extrusion was completed (Figure 5). Then, the newly designed palatal fixed retainer was re-made using 0195 twistflex wire and bonded to all maxillary incisors, including tooth #11 for 6 weeks for stabilization.

Then, teeth #11 and #12 porcelain fused zirconia crowns (zirconium oxide 89.5%, yttrium oxide 7.5%, and vinyl alcohol 3%) were restored under the following procedure. First, the crowns were etched with hydrofluoric acid, and then a silane coupling agent was applied and then air-dried on each crown. The tooth surface was also etched using 37% phosphoric acid and air-dried. Dual-cure resin cement (Duo-Link Universal, BISCO) was used for cementation.

**Figure 6:**
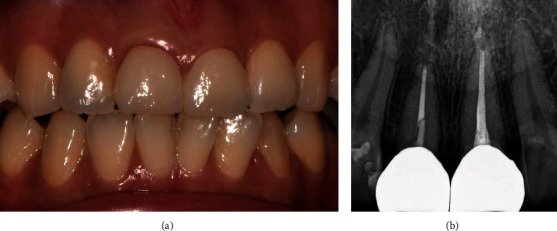
(a) Intra-oral photograph of orthodontic extrusion 6-month follow up. (b) Periapical view of orthodontic extrusion 6-month follow up.

**Figure 7:**
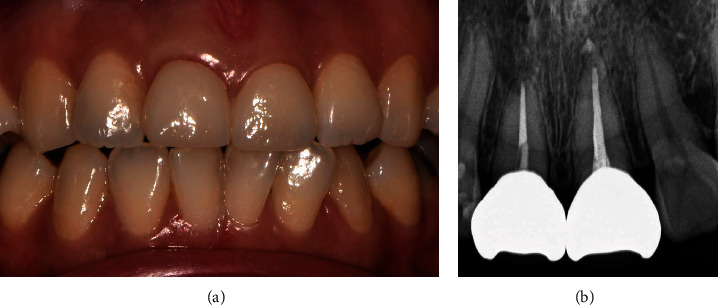
(a) Intra-oral photograph of orthodontic extrusion 15-month follow-up. (b) Periapical view of orthodontic extrusion 15-month follow up.

During 15-month periodic check-ups (Figures 6 and 7) neither specific findings nor complications were observed on each check-up visit. Both the patient and the clinician were satisfied in terms of aesthetics and function.

## 3. Discussion

Orthodontic extrusion, proposed by Heithersay et al. [12] in 1973, refers to a technique, in which a weak and continuous corrective force is applied to move the tooth in a coronal direction. The movement of the shifting tooth causes the gingiva and periodontal zone to stretch, and the soft tissue and bone tissue shift to the dentition. Before orthodontic extrusion, it is important to understand in advance the length, shape, fracture location, importance of the tooth, the patient's age, and the treatment length and cost [13].

Orthodontic extrusion can expose healthy dentin while maintaining biological width so that aesthetically the restorative margins can be properly positioned for a favorable prognosis [14]. At this time, a ferrule of 1.25–2.5 mm is required. Biological width is the sum of epithelial tissue and connective tissue attachment attached to the tooth root above the alveolar ridge (2.04 mm), and the prosthesis margin must be at least 3.5–4 mm away from the alveolar ridge so as not to invade the attachment of the crown [15]. In particular, orthodontic extrusion has been widely performed to ensure biological width in cases where the margins of the teeth are located at the sub-gingival level [16].

The crown-to-root ratio helps predict the prognosis of teeth. The ideal clinical crown-to-root ratio should be 1 : 2, and at least 1 : 1 should be maintained. When orthodontic extrusion was performed, a relatively improved crown-to-root ratio could be obtained compared with surgical treatment [17]. In this case, the length of the crown–root measured from radiographs still maintains a crown-to-root ratio over 1 : 1.5, a more favorable ratio than 1 : 1 after extrusion.

When orthodontic extrusion is performed, the width of the attached gingiva increases, and when the gingival margin moves to the coronal direction, the gingival mucosal border remains the same. By performing periodontal surgery after the extrusion, it can be restored to the aesthetic crown shape in harmony with the adjacent teeth of the maxillary arch [18].

Methods of orthodontic extrusion include slow extrusion using a constant force of 15–30 g and rapid extrusion using a constant force of 50 g or more [8]. In slow extrusion, the movement of the alveolar bone and gingiva occurs at the same time as with tooth extraction, and after orthodontic extrusion, surgical treatment is required to secure biological width [8, 19]. On the contrary, rapid extrusion uses a large static force to move the teeth quickly, so that the dental movement of periodontal tissue does not occur at the same time, reducing the need for additional surgical procedures such as osteotomy [13, 20, 21]. Thus, rapid extrusion is recommended for secure biological width, periodontal ligament fiber expansion, and immediate osteogenesis prevention around the newly formed white enamel border [20].

Orthodontic extrusion can usually be performed using a removable appliance and a rubber band or bracket and wire. However, using a removable appliance needs the patient's degree of cooperation, so it is difficult to use it for certain patients [9]. In addition, there is a possibility that the treatment period may be prolonged due to remanufacturing by device fracture. Next, the method of using fixed wire and bracket attached to 2–4 adjacent teeth, applying a constant force by a power chain or elastic thread, has a limitation of tipping risk of adjacent teeth used as a fixation cannot be avoided. Furthermore, it is unaesthetic due to brackets with difficulty in oral hygiene management. Therefore, in this case, an MTA that can be used for partial orthodontic treatment of the anterior teeth, such as mild diastema and distortion, was used.

By doing so, there is almost no foreign body sensation or pain, and the short correction period (6 weeks as the shortest and 3–6 months average) and the result will also be aesthetically pleasing. In addition, since 012 NiTi wire is used, it has the advantage of less frictional resistance during tooth movement and various side effects, such as root resorption, periodontal damage, and unwanted tooth movement, that may occur during orthodontic treatment [10, 11].

However, commercially available MTAs were expensive, and alternatives were proposed. One example is the method used in the cases above, the use of a syringe tip cut into 3 mm lengths. The process of cutting and polishing the needle so that it can be used as a tube is difficult, but if it is covered with resin, it can maintain its aesthetics, and it is easy to use because it is easy to obtain and affordable.

After the orthodontic extrusion is completed, it is necessary to evaluate whether the traumatic occlusal force is working. In addition, it is necessary to have a sufficient maintenance period. After the orthodontic extrusion is completed, Heithersay and Ingber recommended 2 months and Guilford et al. recommended 3–6 months of maintenance period [22–24]. Still, the maintenance period varies slightly from paper to paper, but the maintenance period of about 2 months is also used for the normal period. Several literature have emphasized the necessity for an incision of the suprcrestal fibers to prevent recurrence and reduce the maintenance period [25–27]. The main periodontal fibers are rearranged during the maintenance period, but the supracrestal fibers remain stretched for a long time, so the excision of these fibers is important to prevent recurrence.

The final result of orthodontic extrusion contributes to more aesthetic and physiological results. The position of the gingival margin is also close to the original level, and the crown-to-root ratio can be relatively improved. This eliminates the non-aesthetic aspects that can be caused by surgical procedures. In this case, a longer follow-up is required for a more accurate prognosis due to the short follow-up period after orthodontic extrusion and methylene blue staining is performed on fractured teeth before orthodontic extrusion to check for crack lines and predict a more accurate prognosis.

Orthodontics is a way to regenerate bone and tissues rather than just extruding the teeth. According to the Paolone et al. study, the orthodontic extrusion procedure can be used for the correction of periodontal defects and the enhancement of the alveolar bone and the soft tissue remodeling [28].

There must be harmony between the soft and hard tissues and between the periodontal of adjacent teeth. Often, a combination of orthodontic extrusion and dental surgery to minimize the need for follow-up treatment for adjacent teeth can improve the crown-to-root ratio and achieve more aesthetic results.

## 4. Conclusion

In this case, orthodontic extrusion was used for complex crown–root fracture of maxillary anterior teeth due to trauma to secure a relatively sufficient clinical crown length. When orthodontic extrusion using customized MTA was performed, it was possible to improve the crown-to-tooth root ratio compared with surgical treatment, and it is considered to be a satisfactory and effective treatment method for both patients and practitioners in terms of short treatment duration and aesthetics.

## Data Availability

Data supporting this research article are available from the corresponding author or first author upon reasonable request.

## References

[1] Tapias M. A., Jimenez-Garcia R., Lamas F., Gil A. A. (2003). Prevalence of traumatic crown fractures to permanent incisors in a childhood population: Móstoles, Spain. *Dental Traumatology*.

[2] Dede D. O., Tunc E. S., Guler A. U., Yazicioğlu S. (2017). Multidisciplinary approach to a subgingivally fractured incisor tooth: a case report. *The Journal of Dental Sciences*.

[3] Koyuturk A. E., Malkoc S. (2005). Orthodontic extrusion of subgingivally fractured incisor before restoration. A case report: 3-years follow-up. *Dental Traumatology*.

[4] Gupta G., Gupta R., Gupta N., Gupta U. (2015). Crown lengthening procedures- a review article. *IOSR Journal of Dental and Medical Sciences*.

[5] Nugala B., Kumar B. S., Sahitya S., Krishna P. M. (2012). Biologic width and its importance in periodontal and restorative dentistry. *Journal of Conservative Dentistry*.

[6] Fidel S. R., Fidel-Junior R. A., Sassone L. M., Murad C. F., Fidel R. A. (2011). Clinical management of a complicated crown-root fracture: a case report. *Brazilian Dental Journal*.

[7] Saito C. T., Guskuma M. H., Gulinelli J. L. (2009). Management of a complicated crown-root fracture using adhesive fragment reattachment and orthodontic extrusion. *Dental Traumatology*.

[8] Mittal R., Gupta S., Singla A., Gupta A. (2013). Managing subgingival fracture by multi-disciplinary approach: endodontics-forced orthodontic extrusion and prosthetic rehabilitation. *Saudi Endodontic Journal*.

[9] Mandel R. C., Binzer W. C., Withers J. A. (1982). Forced eruption in restoring severely fractured teeth using removable orthodontic appliances. *The Journal of Prosthetic Dentistry*.

[10] Hwang H. S., Jeon H. R., Kim S. P., Kim W. S., Lee G. H. (2011). A new orthodontic appliance for rapid anterior alignment in adults; mini-tube appliance (MTA). *Journal of the Korean Dental Association*.

[11] Kim H. I., Lim S. H., Gang S. N. (2016). Orthodontic treatment using mini-tube appliances and customized resin domes using 3D CAD design. *Oral Biology Research*.

[12] Ziskind D., Schmidt A., Hirschfeld Z. (1998). Forced eruption technique: rationale and clinical report. *The Journal of Prosthetic Dentistry*.

[13] Reitan K. (1967). Clinical and histologic observations on tooth movement during and after orthodontic treatment. *American Journal of Orthodontics*.

[14] Emerich-Poplatek K., Sawicki L., Bodal M., Adamowicz-Klepalska B. (2005). Forced eruption after crown/root fracture with a simple and aesthetic method using the fractured crown. *Dental Traumatology*.

[15] Khuller N., Sharma N. (2009). Biologic width: evaluation and correction of its violation. *Journal of Oral Health and Community Dentistry*.

[16] Stern N., Becker A. (1980). Forced eruption: biological and clinical considerations. *Journal of Oral Rehabilitation*.

[17] Potashnick S., Rosenberg E. S. (1982). Forced eruption: principles in periodontics and restorative dentistry. *The Journal of Prosthetic Dentistry*.

[18] Kwon E. Y., Lee J. Y., Choi J. (2016). Effect of slow forced eruption on the vertical levels of the interproximal bone and papilla and the width of the alveolar ridge. *Korean Journal of Orthodontics*.

[19] Bach N., Baylard J. F., Voyer R. (2004). Orthodontic extrusion: periodontal considerations and applications. *Journal of the Canadian Dental Association*.

[20] Bondemark L., Kurol J., Hallonsten A. L., Andreasen J. O. (1997). Attractive magnets for orthodontic extrusion of crown-root fractured teeth. *American Journal of Orthodontics and Dentofacial Orthopedics*.

[21] Zyskind K., Zyskind D., Soskolne W. A., Harary D. (1992). Orthodontic forced eruption: case report of an alternative treatment for subgingivally fractured young permanent incisors. *Quintessence International*.

[22] Heithersay G. S. (1973). Combined endodontic-orthodontic treatment of transverse root fractures in the region of the alveolar crest. *Oral Surgery, Oral Medicine, and Oral Pathology*.

[23] Ingber J. S. (1974). Forced eruption: Part I. A method of treating isolated one and two wall infrabony osseous defects - rationale and case report. *Journal of Periodontology*.

[24] Guilford H. J., Grubb T. A., Pence D. L. (1984). Vertical extrusion: a standardized technique. *The Compendium of Continuing Education in Dentistry*.

[25] Kozlovsky A., Tal H., Lieberman M. (1988). Forced eruption combined with gingival fiberotomy. A technique for clinical crown lengthening. *Journal of Clinical Periodontology*.

[26] Shyammohan A. (2011). Forced eruption: an adjunct to prosthodontic treatment planning. *Indian Journal of Stomatology*.

[27] Bajaj P., Chordiya R., Rudagi K., Patil N. (2015). Multidisciplinary approach to the management of complicated crown-root fracture: a case report. *Journal of International Oral Health*.

[28] Paolone M. G., Kaitsas R., Palolone G., Kaitsas V. (2008). Lingual orthodontics and forced eruption: a means for osseous and tissue regeneration. *Progress in Orthodontics*.

[29] Lang N. P., Löe H. (1972). The relationship between the width of keratinized gingiva and gingival health. *Journal of Periodontology*.

